# Neuromuscular monitoring and incidence of postoperative residual neuromuscular blockade: a prospective observational study

**DOI:** 10.1186/s44158-025-00226-1

**Published:** 2025-01-28

**Authors:** Alessandra Piersanti, Rossella Garra, Fabio Sbaraglia, Miryam Del Vicario, Rosa Lamacchia, Marco Rossi

**Affiliations:** Department of Anesthesia and Intensive Care, Agostino Gemelli IRCCS University Polyclinic Foundation, Rome, Italy

**Keywords:** NMBA, Neuromuscular monitoring, RNMB, Residual neuromuscular blockade, Residual paralysis, Respiratory complications

## Abstract

**Background:**

Neuromuscular blocking agents (NMBAs) are routinely used in anesthesia practice. An undetected, incomplete recovery of neuromuscular function at the end of surgery potentially exposes patients to clinical deterioration in the postoperative period.

The aim of this study was to investigate the incidence of postoperative residual neuromuscular blockade (RNMB) in a cohort of patients receiving NMBAs.

**Methods:**

We enrolled 90 spontaneously breathing adult patients admitted to the recovery room (RR) after completion of surgeries having received at least 1 dose of NMBA. Anesthesia management, the dosage of NMBA used, and whether monitoring of neuromonitoring function was employed or if a reversal agent was administered were all at the discretion of the attending anesthesiologist, who was unaware that neuromuscular function was going to be monitored in the RR.

The primary objective of this study was to determine the incidence of RNMB (defined as a train-of-four ratio ≤ 0.9). The secondary objectives were the number of postoperative adverse respiratory events and, for exploratory purposes, the estimation of potential risk factors through logistic regression analysis.

**Results:**

RNMB occurred in 5 (5%) patients who had received only one dose of NMBA at induction of anesthesia.

Two patients with RNMB (40%) required O_2_ supplementation during monitoring in the RR, compared to 11 patients in the rest of the sample (13%). Additionally, 2 of these patients (2%) required O_2_ supplementation before hospital discharge due to O_2_ desaturation < 92%. None of the patients with RNMB had received a reversal of neuromuscular blockade at the end of surgery.

The association between RNMB and potential risk factors, assessed through multivariable logistic regression did not yield significant results for any of the considered variables.

**Conclusions:**

RNMB can occur even when a single dose of NMBAs is administered. Despite decades of extensive literature on the risks of RNMB and recent guidelines, routine monitoring of neuromuscular function and pharmacologic reversal of NMBA is still substandard. Routine monitoring of neuromuscular function is strongly advocated to enhance the level of patient care.

**Trial registration:**

The study was registered at ClinicalTrials.gov (NCT06193213, date of registration: 05/01/2024).

**Supplementary Information:**

The online version contains supplementary material available at 10.1186/s44158-025-00226-1.

## Introduction

Neuromuscular blocking agents (NMBAs) are routinely used in anesthesia practice. An undetected, incomplete recovery of neuromuscular function at the end of surgery exposes patients to potential and preventable postoperative adverse respiratory events [1, 2].

To date, real-time measurement of the train-of-four ratio (TOFR) at the level of the adductor pollicis muscle is considered the most appropriate method for neuromuscular assessment in clinical practice. Despite the recognition of quantitative neuromuscular monitoring (NMM) as the only effective measure for avoiding or reducing postoperative residual neuromuscular blockade (RNMB), the incidence of RNMB due to inadequate NMM is still high [3, 4].

Both the literature and expert opinion have ascribed substandard NMM to the limited availability of monitoring devices in some hospitals and to clinicians’ reluctance, attributable either to the belief that RNMB is a rare event or overconfidence in their ability to qualitatively assess patient’s neuromuscular function recovery [5].

The primary objective of this study was the incidence of RNMB in patients admitted to the recovery room (RR) after the completion of surgeries of any duration involving the use of nondepolarizing intermediate-acting neuromuscular blockers. Residual paralysis was defined as a TOFR ≤ 0.9.

The secondary objectives were the number of postoperative adverse respiratory events and for exploratory purposes, the estimation of potential risk factors through logistic regression analysis.

## Methods

This observational prospective study adheres to the applicable Strengthening the Reporting of Observational Studies in Epidemiology (STROBE) guidelines. It was approved by the Territorial Ethics Committee (Comitato Etico Territoriale Lazio Area 3–Roma, ID Number: 5991, Protocol Number 0000494/23, 29/11/2023) and it was registered at ClinicalTrials.gov (NCT06193213, date of registration: 05/01/2024).

Written, informed consent was obtained from all patients before enrolment. The study was conducted between February and May 2024 at Fondazione Policlinico Universitario Agostino Gemelli IRCSS, Rome, Italy, in accordance with Good Clinical Practice guidelines and the principles of the Declaration of Helsinki.

We included 90 spontaneously breathing adult patients, American Society of Anesthesiologists (ASA) physical status I-III, who were admitted to the RR after completion of non-abdominal surgery under general anesthesia having received at least one dose of a nondepolarizing neuromuscular blocking agent for endotracheal intubation or maintenance of neuromuscular blockade.

Patients undergoing emergency surgery, those with neuromuscular pathologies, or those requiring postoperative monitoring in the Intensive Care Unit were excluded from the study.

Given the observational nature of the study, anesthesia management was not standardized—dosing of NMBAs, use of NMM, administration of reversal agents, and timing of extubation were all left to the discretion of the attending anesthesiologist, in accordance with its clinical routine. The anesthesiologist in charge in the operating room was unaware that neuromuscular function was going to be monitored in the RR.

NMM devices available in every operating theatre are TOFscan® Dräger-Drägerwerk AG & Co. KGaA, Germany or NMT MechanoSensor, GE Healthcare–United States.

Within 5 min from arrival in the RR, an independent researcher assessed the contraction of the adductor pollicis using acceleromyography (TOFscan® Dräger-Drägerwerk AG & Co. KGaA, Germany).

Two TOFR measurements were performed 30 s apart. The nerve was stimulated with 4 stimuli of 40 mA of amplitude, at a frequency of 2 Hz, each of a duration of 0.2 ms. In analogy with other studies [6, 7], if the difference between the two measurements was ≤ 10%, the average value was considered for the analysis. If the difference was > 10%, a third measurement was taken, and the two closest results were averaged. Those patients presenting with residual blockade were monitored and eventually administered sugammadex to restore normal neuromuscular function, assessed by subsequent TOFR measurements.

Data related to the anesthesia performed, including the agents used, whether neuromuscular monitoring was employed, and the timing of administration of neuromuscular blocking drugs and reversal agents, if applicable, were recorded. The duration of stay in the RR along with any adverse respiratory events that occurred in the RR and up until hospital discharge were also documented. Respiratory adverse events were defined as episodes of desaturation (SpO_2_ < 92%) requiring oxygen supplementation or the detection of atelectasis, pneumonia, or pleural effusion of noncardiac origin found on thoracic imaging tests eventually performed during the hospital stay.

## Statistical analysis

From a meta-analysis published in 2020 by Carvalho [8], which included 53 studies and a total of 12,664 patients, we extrapolated 22 studies (*n* = 4268) with population, objectives, and methodology similar to our study. In these studies, regardless of whether an intraoperative monitoring system of neuromuscular function was adopted, the incidence of RNMB (defined as a TOFR ≤ 0.9) after the use of intermediate-acting neuromuscular blockers, detected in the RR using acceleromyography was 28%.

Assuming the same incidence of 28%, we estimated that a sample size of 78 patients would be required to estimate the expected proportion with an absolute precision of 10% and a 95% confidence level.

Assuming a drop-out of 15%, the sample was increased to 90 patients.

Data are presented as mean ± standard deviation or median (interquartile range) for numerical data or *N* (%) for categorical or ordinal data. The normality distribution of numerical data was assessed with the Shapiro–Wilk test and visually by histograms.

The association between RNMB and potential risk factors was assessed for exploratory purposes using multivariable logistic regression analysis. Patient age, duration of anesthesia, type of anesthesia (inhalational or total intravenous), type of NMBA, number of doses and total dosage of NMBA administered, and whether or not neuromuscular blockade was reversed were considered as possible risk factors. Multicollinearity was assessed by calculating variance inflation factors (VIF) for each considered variable.

A *p-*value < 0.05 was considered statistically significant. Data analysis was performed using *R* (R Foundation for Statistical Computing, Austria; version 4.3.3).

## Results

Ninety patients were included in this analysis. The mean age was 57 (42, 71) years, and the majority were female (52%) and classified as ASA II (60) (see Table 1).

**Table 1:** Patient characteristics

Patient characteristics	Total patients (*N* = 90)
Age, years	57 (42, 71)
Height, cm	168 ± 9
Weight, kg	73 ± 13
Body mass index, kg/m^2^	26 ± 4
Female	47 (52)
Male	43 (48)
ASA status	
1	27 (30)
2	54 (60)
3	9 (10)
Comorbidities	
Cardiovascular diseases	33 (37)
Pulmonary diseases	11 (12)
Diabetes	6 (7)
Renal	1 (1)
History of cancer	15 (17)
Type of surgery	
Head and neck	45 (50)
Spinal	28 (31)
Breast	7 (8)
Reconstructive	7 (8)
Orthopedic	3 (3)

Data are presented as *N* (%), mean ± standard deviation, or median (interquartile range)

*ASA* American Society of Anesthesiologists

The most common type of surgery was head and neck followed by spinal surgery, with a median anesthesia duration of 121 (79, 215) min.

Rocuronium was the sole NMBA used and only 5 (5%) patients received more than 1 dose during surgery. The mean administered dose of NMBA was 0.5 (0.20) mg kg^−1^. Neuromuscular monitoring was documented on the anesthesia charts for 41 patients (45%), and TOFR at extubation was recorded for 24 patients (27%) (Table 2).

**Table 2:** Perioperative data and results

Perioperative data and results	Total patients (*N* = 90)
Duration of surgery, min	87 (58, 170)
Duration of anesthesia, min	121 (79, 215)
Time from last NMBA dose administration to the end of anesthesia in patients who received > 1 dose, min	167 (115, 185)
Time from end of anesthesia to arrival in the RR, min	6 (5, 8)
Inhalational anesthesia	51 (57)
Target-controlled infusion anesthesia	39 (43)
Total dosage of sufentanil, mcg	15 (10, 20)
Total dosage of fentanyl, mcg	200 (100, 200)
Use of remifentanil	52 (58)
Neuromuscular blocking agent:	
Rocuronium	90 (100)
Total dosage of rocuronium, mg kg^−1^	0.50 (0.20)x\x\
Received ≥ 1 dose of rocuronium	5 (5)
Reversal agent use:	
Sugammadex	11 (12)
Total dose of sugammadex, mg	200 (200, 300)
Number of anesthesia monitoring charts that reported intraoperative neuromuscular monitoring	41 (45)
Number of anesthesia monitoring charts that reported basal TOFR at induction of anesthesia	3 (3)
Number of anesthesia monitoring charts that reported TOFR at extubation	24 (27)
Incidence of TOFR ≤ 0.9 at arrival in the RR	5 (5)
Patients with TOFR ≤ 0.9 requiring O_2_ supplementation during recovery room stay	2 (2)
Monitoring time in the RR, min	60 (45, 75)
Hospital length of stay, days	2 (1, 3)

Data are presented as *N* (%), mean ± standard deviation or median (interquartile range)

*NMBA* neuromuscular blocking agent, *TOFR* train-of-four ratio

Baseline TOFR was reported for only 3 patients so that normalized TOFR could not be evaluated for patients whose neuromuscular function was assessed with GE devices (NMT MechanoSensor, GE Healthcare–United States). Sugammadex was used to reverse residual neuromuscular blockade in 11 patients (12%) at the end of surgery.

RNMB occurred in 5 (5%) patients upon arrival in the RR. Three of them underwent head or neck surgery and the other two patients, spinal surgery. The mean duration of anesthesia was 136 (59) min. All of them had received only 1 dose of NMBA at induction of anesthesia and TOFR at the end of surgery was not documented on the anesthesia monitoring chart for any of these patients.

Two patients with RNMB (40%) required O_2_ supplementation during monitoring in the RR, compared to 11 patients (13%) in the rest of the sample. Additionally, 2 of these 11 patients (2%) required O_2_ supplementation before hospital discharge due to O_2_ desaturation < 92%. None of patients with RNMB had received a reversal of neuromuscular blockade at the end of surgery and all of them had received only one dose of NMBA.

The association between RNMB and related potential risk factors, assessed through multivariable logistic regression did not yield significant results for any considered variable (see Table 3 in Supplementary Materials).

## Discussion

The main findings of the present study are that RNMB upon arrival in RR was not trivial (5%) even if only 5/90 patients had received more than one dose of NMBA during surgery; intraoperative NMM rate was low (45% of total enrolled cases), TOFR before extubation even lower (27%).

In most procedures performed in our specific setting, muscle paralysis is induced to improve intubation quality, whilst deep NMB during maintenance of anesthesia is rarely needed.

Among other factors, age ≥ 65 years [9], possibly due to slower drug metabolism, and female sex [10, 11] have been associated with RNMB when a single dose of NMBA was administered to all or the vast majority of patients.

A long interval between the last administration of muscle relaxant and extubation does not guarantee full recovery, as a wide interindividual variability in the duration of action of NMBAs might exist [12]. Our findings further confirm those from a previous study from Debaene et al. [13], who, in a cohort of 238 patients, found that residual paralysis, tested more than 2 h after NMBA administration, had an incidence of 37%.

National and international guidelines from anesthesiologist societies [1, 4, 12, 14–17], expert opinions, and review articles all advocate for the use of quantitative monitoring of neuromuscular function whenever a muscle relaxant is used, as an effective measure to limit the risk of postoperative respiratory complications. However, evidence shows that compliance by anesthesiologists is low. A recent survey on neuromuscular blockade management in Europe showed that on a total of around 17,000 patients exposed to a NMBA, NMM was not used in nearly 60% of cases, which unsurprisingly increased the rate of relaxant-associated postoperative pulmonary complications [18].

In our observational study, NMM was documented to have been adopted for only the 45% of the patients and this percentage is in line with a comprehensive meta-analysis published in 2020 by Carvalho [8], which reported the use of quantitative monitoring in 48% of 12,664 patients.

The erroneous belief that residual neuromuscular blockade is a rare event, the limited availability or malfunction of NMM equipment in some hospitals, patient’s position, time pressure, lack of training, department culture [19], and a significant degree of overconfidence among anesthesiologists in their ability to manage NMBA use without such guidance, may partially explain the failure of its routinary adoption [20].

To improve the level of patient care, a number of organizational and cultural changes have been proposed by recent literature [4, 19] including widespread adoption of NMM devices (preferably integrated into anesthesia machines in every operating theatre), standardized monitoring and reporting routines across workplaces to maintain consistency in resident training, enhanced medical education with e-learning modules and decision algorithms, proper use, timing and dosages of reversal agents, departmental tools like checklists to track guidelines adherence and an-interned based reporting site intended to identify the main obstacles to implementation.

Two out of 5 patients with RNMB received sevoflurane for anesthesia maintenance: even if it is known that inhalation agents prolong neuromuscular block, exploratory results of logistic regression as well as proportions of patients with RNMB depending on the type of anesthesia did not yield statistically significant results (Fisher’s exact test *p* = 0.651). Despite the limited sample size, these results are consistent with those of Naguib [21] and Carvalho [8].

In our surgical block, sugammadex was the preferred agent for reversing NMBA. This preference can be attributed to the indirect mechanisms of action of anticholinesterase inhibitors, such as neostigmine, which are associated with limited and unpredictable efficacy, ceiling effect, as well as undesirable autonomic side effects, including hypersalivation, bradycardia, bronchoconstriction, and nausea [22].

Interestingly, Hayes et al. [9] in 2001 found that of 101/148 subjects who had received reversal with neostigmine, 49 (49%) had shown postoperative RNMB.

Recently, a Cochrane systematic review and meta-analysis [23] reported faster reversal times regardless of the depth of NMB, and a better safety profile for sugammadex compared to neostigmine.

This analysis is based on the results of an observational study of a limited sample size, and it should be interpreted in light of its limitations. It was designed to reflect current practice, so the presence of possible biases cannot be excluded.

For instance, neuromuscular function monitoring was recorded on anesthesia sheets in less than half of the cases, but we cannot exclude that NMM had been adopted during surgery but not reported.

However, no serious postoperative respiratory events were observed. Two patients with RNMB (40%) required O_2_ supplementation during monitoring in the RR, compared to 11 patients (13%) in the rest of the sample, and 2 (2%) of them also required oxygen supplementation before hospital discharge due to O_2_ desaturation < 92%.

Mechanisms other than residual neuromuscular blockade, such as atelectasis induced by general anesthesia itself, concomitant opioid use, or patient-specific risk factors, may have contributed to the deterioration of gas exchange in the postoperative period [24].

It is worth noting that most of the patients enrolled were ASA I–II. We can hypothesize that more severe effects might have been observed in frailer cases, where even a limited desaturation could increase the risk of serious consequences.

None of the patients who experienced minor respiratory events had received NMBA reversal at the end of surgery.

A previous study by Martinez-Ubieto and collegues [25] found that the global incidence of minor respiratory episodes could be lowered by reversing neuromuscular blockade at the end of surgery.

## Conclusions

Despite decades of extensive literature on the risks of RNMB and guidelines on perioperative management of neuromuscular blockade, NMM remains far from being routine practice. This study reinforces the observation that current practice is inadequate and RNMB can occur even after the administration of a single dose of NMBA.

Routine monitoring of neuromuscular function is strongly advocated to improve the quality of patient care, enhance recovery after surgery, and reduce postoperative complications, but this requires both organizational and individual behavioral changes.

## Supplementary Information

Additional file 1. Table 3 in supplementary materials.

## Data Availability

Datasets generated and/or analyzed during this study are available from the corresponding author upon reasonable request.

## Abbreviations

NMBAs: Neuromuscular blocking agents
RNMB: Residual neuromuscular blockade
TOFR: Train-of-four ratio
NMM: Neuromuscular monitoring
RR: Recovery room
STROBE: Strengthening the reporting of observational studies in epidemiology
ASA: American Society of Anesthesiologists
