# Supplementary material for: Neuromuscular monitoring and incidence of postoperative residual neuromuscular blockade: a prospective observational study
Source: J Anesth Analg Crit Care. 2025 Jan 28;5:5. doi: 10.1186/s44158-025-00226-1 (PMC11776315; doi:10.1186/s44158-025-00226-1)
Supplement: Supplementary file 1 — Additional file 1. Table 3 in supplementary materials. [file 44158_2025_226_MOESM1_ESM.docx]

| **Variable** | **Estimate** | **Std. Error** | **z value** | **Pr(>\|z\|)** | **Pr(>Chi)** | **VIF values** |
| --- | --- | --- | --- | --- | --- | --- |
| (Intercept) | 10.861 | 4.134e+03 | 0.003 | 0.998 |  |  |
| Age | 0.037 | 2.938e-02 | 1.282 | 0.200 | 0.248 | 1.043 |
| Duration of anesthesia | -0.002 | 6.192e-03 | -0.467 | 0.641 | 0.653 | 1.097 |
| Type of anesthesia (inhalational or total intravenous | -1.220 | 1.011e+00 | -1.208 | 0.227 | 0.223 | 1.080 |
| Number of doses of NMBA administered | -15.711 | 4.134e+03 | -0.004 | 0.997 | 0.996 | 1.000 |
| Total dosage of administered NMBA | 0.031 | 3.247e-02 | 0.979 | 0.328 | 0.406 | 1.095 |
| Reversal of NMBA | -18.041 | 5.003e+03 | -0.004 | 0.997 | 0.202 | 1.000 |
|  |  |  |  |  |  |  |

**Table 3**: Results of logistic regression model, including coefficients associated to each variable (Estimate column), standard error (Std Error column), log odds (z value column), P value associated to the z value (Pr(>|z| column), P value associated to Chi-square test and VIF (variance inflation factors) values.
